# Non-Invasive Method to Detect Infection with *Mycobacterium tuberculosis* Complex in Wild Boar by Measurement of Volatile Organic Compounds Obtained from Feces with an Electronic Nose System

**DOI:** 10.3390/s21020584

**Published:** 2021-01-15

**Authors:** Kelvin de Jesús Beleño-Sáenz, Juan Martín Cáceres-Tarazona, Pauline Nol, Aylen Lisset Jaimes-Mogollón, Oscar Eduardo Gualdrón-Guerrero, Cristhian Manuel Durán-Acevedo, Jose Angel Barasona, Joaquin Vicente, María José Torres, Tesfalem Geremariam Welearegay, Lars Österlund, Jack Rhyan, Radu Ionescu

**Affiliations:** 1 Mechatronics Engineering Department, Universidad Autónoma del Caribe, Barranquilla 080020, Colombia; kelvin.beleno@uac.edu.co; 2 GISM Group, Faculty of Engineering and Architecture, University of Pamplona, Pamplona 543050, Colombia; juan.caceres@unipamplona.edu.co (J.M.C.-T.); lisset.jaimes@unipamplona.edu.co (A.L.J.-M.); oscar.gualdron@unipamplona.edu.co (O.E.G.-G.); cmduran@unipamplona.edu.co (C.M.D.-A.); 3 Centers for Epidemiology and Animal Health, Veterinary Services, Animal and Plant Health Inspection, Service, United States Department of Agriculture, Fort Collins, CO 80526, USA; pauline.nol@state.co.us (P.N.); rhyanjack@yahoo.com (J.R.); 4 VISAVET Health Surveillance Centre, Animal Health Department, Faculty of Veterinary Medicine, Complutense University of Madrid, 28040 Madrid, Spain; jbarason@ucm.es; 5 SaBio Instituto de Investigación en Recursos Cinegéticos IREC, ETSIA Ciudad Real, University of Castilla La Mancha & CSIC, 13003 Ciudad Real, Spain; joaquin.vicente@uclm.es; 6 Biomedical Institute of Sevilla (IBiS), University of Seville, University Hospital Virgen del Rocío/CSIC, 41071 Seville, Spain; mjtorres@us.es; 7 The Ångström Laboratory, Department of Materials Science and Engineering Sciences, Uppsala University, P.O. Box 35, 75103 Uppsala, Sweden; tesfalem.welearegay@angstrom.uu.se (T.G.W.); lars.osterlund@angstrom.uu.se (L.Ö.); 8 Institute of Veterinary Medicine and Animal Sciences, Estonian University of Life Sciences, 51006 Tartu, Estonia

**Keywords:** *Sus scrofa*, bovine tuberculosis, *Mycobacterium bovis*, diagnosis, feces, volatile organic compounds, chemical gas sensors, gold nanoparticles, organic ligands

## Abstract

More effective methods to detect bovine tuberculosis, caused by *Mycobacterium bovis*, in wildlife, is of paramount importance for preventing disease spread to other wild animals, livestock, and human beings. In this study, we analyzed the volatile organic compounds emitted by fecal samples collected from free-ranging wild boar captured in Doñana National Park, Spain, with an electronic nose system based on organically-functionalized gold nanoparticles. The animals were separated by the age group for performing the analysis. Adult (>24 months) and sub-adult (12–24 months) animals were anesthetized before sample collection, whereas the juvenile (<12 months) animals were manually restrained while collecting the sample. Good accuracy was obtained for the adult and sub-adult classification models: 100% during the training phase and 88.9% during the testing phase for the adult animals, and 100% during both the training and testing phase for the sub-adult animals, respectively. The results obtained could be important for the further development of a non-invasive and less expensive detection method of bovine tuberculosis in wildlife populations.

## 1. Introduction

Bovine tuberculosis (bTB), caused by *Mycobacterium bovis (M. bovis)*, is an emerging disease among wild animals in many parts of the world, and threatens to spill back as a potential source of infection for livestock and humans [1]. bTB can be easily transmitted from an infected animal to a non-infected one with whom it shares the same habitat, prevailing the aerogenic transmission. It can also be transmitted from animals to humans through consumption of meat products of infected animals, by contact with the blood of infected animals during slaughter, or through inhalation of air exhaled by infected animals [2,3]. In humans, bTB causes severe morbidity and even death, especially in the case of people involved in hunting and slaughter of animals without sufficient sanitary control.

Standard bTB testing in live animals consists of single intradermal tuberculin tests for screening (caudal fold test or single cervical skin test) with a comparative cervical test, or an interferon gamma release assay test, as supplemental or confirmatory tests [4,5,6]. The final diagnosis of bovine tuberculosis requires post-mortem laboratory confirmation of the disease via histopathology, polymerase chain reaction, and bacteriological culture [5,6].

The detection of bovine tuberculosis in wildlife is generally done through techniques such as hunter-kill surveys, road-kill surveys, or actively capturing and/or killing animals for serologic testing and/or post-mortem examination [7,8,9]. There is need for less invasive, less expensive techniques to remotely detect bTB disease in wildlife populations.

The detection of disease-specific volatile organic compounds (VOCs) from the feces may provide a solution for remote surveillance of wildlife. They reflect metabolomic changes produced by the disease in the host, and their analysis emerged as a promising approach for disease diagnosis and monitoring [10].

Two complementary methods are generally applied for VOC analysis: analytical techniques and electronic nose systems. The analytical techniques allow for the identification of disease-specific biomarkers. Yet, the concentration of many compounds emitted by biological samples is below the limit of detection of the analytical equipment and not all possible biomarkers are detected [11]. The electronic nose systems are formed of an array of cross-reactive chemical gas sensors, where each sensor lacks sufficient selectivity for detecting individual VOCs in a gaseous mixture, but, instead, the electronic nose system is trained to detect a disease-specific VOC pattern [12]. The electronic nose systems present advantages such as portability, easy operation, easy interpretation of results, and low cost [12].

Apparently, unique VOCs or patterns of VOCs were detected through analytical studies in the feces of cattle and white-tailed deer infected with *M. bovis* [13,14], and in the feces of goats infected with *Mycobacterium avium* paratuberculosis [15,16]. Very recently, in the first study of this kind performed on wild boar (*Sus scrofa*), we reported the identification of *M. bovis*-specific VOC patterns in the feces and breath of wild boar [17]. Similarly, electronic nose systems were also shown to be able to detect the VOC pattern of *M. bovis* in the headspace of serum from cattle and badgers [18,19], and in the breath of cattle [20].

In the present study, we report for the first time the detection of *M. bovis* from the measurement of the VOCs emitted from feces with an electronic nose system. This study was performed on free-ranging wild boar living in Doñana National Park, Spain, where bTB is endemic in the ungulate host community, including wild boar [21]. The successful development of this technology could lead to devices designed to remotely and non-invasively detect *M. bovis* and other pathogens in wildlife populations.

## 2. Materials and Methods

### 2.1. Animals and Samples

The realization of this study was approved by the Commission of Animal Experiments from the University of Castilla-La Mancha, Spain (PR-2015-03-08). The procedures that were applied in this study were designed and implemented by scientists with experience in the field, after approval from the Spanish Ethics Committee in accordance with the European Commission Directive 86/609/EEC for animal experiments.

Fecal samples were obtained from 37 wild boars that were captured in Doñana National Park, Spain, as described in Nol et al. [17] and Barasona et al. [22]. The animals were captured at different sites from the park, located on a radius of 4.4 km, including Martinazo, Palacio, Santa Olalla, and Fuente del Duque. Boar were trapped in a portable cage and corral traps, as described in Barasona et al. [14]. Age determination was based on the recommendations given in Sáenz de Buruaga et al. [23]. Sub-adult and adult animals (12–24 months and >24 months, respectively) were sedated using a combination of tiletamine-zolazepam (100 mg/mL Zoletil^®^, Virbac, Carros, France, 3 mg/kg dose) and medetomidine (Medetor^®^, Virbac, Carros, France, 0.05 mg/kg dose), intramuscularly administered in the lateral region of animal’s gluteus with 5-mL darts (Telinject^®^, Römerberg, Germany) using a 14-mm diameter blowpipe (Telinject^®^, Römerberg, Germany) [22]. Juvenile animals (<12 months) were manually restrained.

Fecal samples were collected from all animals, either manually per rectum or postmortem when no feces could be obtained per rectum during live animal handling. Volatile organic compounds were collected from the VOC headspace formed by approximately 5 g of a fecal sample inside a 125-mL glass jar, as described by Nol et al. [9], via a vacuum pump (AirChek XR5000, SKC Inc., Eighty-Four, PA, USA) into a glass cartridge containing sorbent material suitable for VOC preconcentration and storing (Tenax TA, Sigma Aldrich, St. Louis, MO, USA). The samples were stored at 4 °C before analysis.

Once the fecal samples were collected, the animals were euthanized via captive bolt and exsanguination as part of Doñana National Park population control and health-monitoring program. The wild boars were necropsied, and blood, lung lobes, lymph nodes, and any other tissue displaying lesions compatible with *M. bovis* infection were cultured and analyzed for identification of *M. bovis*, as described by Nol et al. [9] and Safianowska et al. [24].

Full characterization of the animals included in this study and number of fecal VOC samples analyzed per animal are presented in Table 1. Animal grouping on age, disease, sex, and location is summarized in Table 2.

### 2.2. Electronic Nose and Sensing Measurements

**Figure 1:**
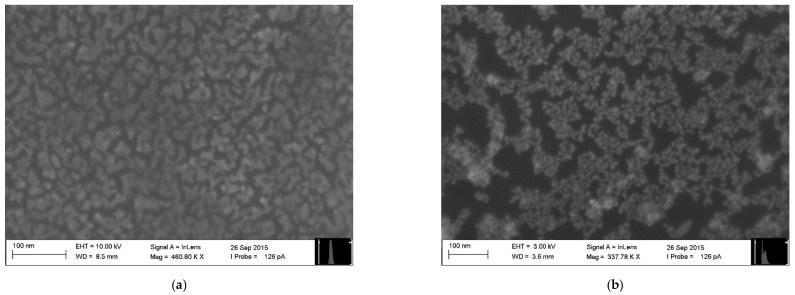
Surface Electron Microscopy (SEM) images of gold nanoparticles functionalized with: (**a**) 2-Mercaptobenzoxazole and (**b**) 4-Methoxy-α-toluenethiol.

The electronic nose employed in this study consisted of 10 chemical gas sensors based on monolayers of ultrapure organically-functionalized gold nanoparticles fabricated employing the Advanced Gas Deposition (AGD) technique, as described in Welearegay et al. [25]. The sensors were fabricated on 13 × 8 mm^2^ one-side polished p-type silicon substrates provided with two parallel gold electrodes on their polished side, forming a 15-μm-gap active area region, where the sensing material was deposited. The characteristics of the sensors with different organic functionalities are presented in Table 3. Differences in their resistance are due to variations of the Au nanoparticle assembly structure and conductivity of the cross-linked functional organic groups attached to the Au nanoparticles. For the sake of exemplification, two sensing nanomaterials are presented in Figure 1.

**Figure 2:**
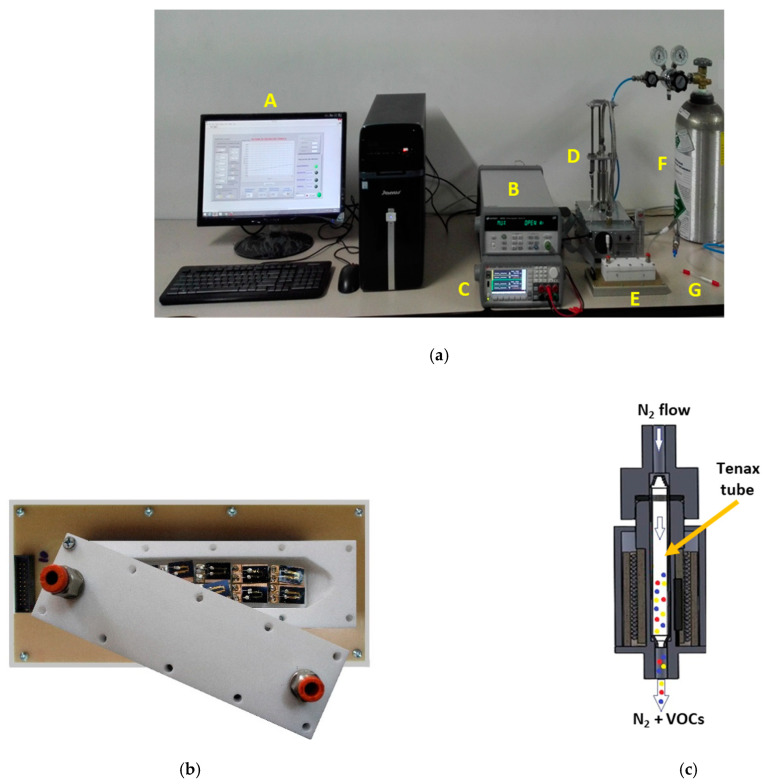
(**a**) Measurement setup: A—computer, B—data acquisition system, C—power source, D—thermal desorption unit, E—sensor test chamber, F—N_2_ gas bottle, and G—Tenax TA storage tube. (**b**) Inner view of the sensor test chamber with the chemical gas sensors. (**c**) Schematic representation of the thermal desorption unit, illustrating released fecal VOCs transported by the N_2_ gas flow.

The setup employed for performing the measurement of the fecal VOC samples with the electronic nose system is shown in Figure 2. The sensors were placed inside a 26-cm^3^ inner volume Teflon test chamber provided with two openings for the sample inlet and sample outlet, respectively (Figure 2a(E),b). The Tenax TA sorbent tube storing the fecal VOC sample (Figure 2a(G)) was heated at 250 °C for 5 min inside a home-built temperature-controlled thermal desorption unit (Figure 2a(D),c) to desorb the VOCs from the Tenax TA matrix.

Sample measurements comprised the following cycles.

(i) 5 min of continuous N_2_ flow (delivered from a commercial N_2_ gas bottle, Cryogas S.A., Colombia—Figure 2a(F)) passed at 5 L/min flow rate through the sensor test chamber for purging purposes before the sample measurement,
(ii) 5 min of exposure to the fecal VOCs carried by continuous N_2_ flow that passed at 100 mL/min flow rate at first through the thermal desorption unit for taking the thermally released VOCs (see Figure 2c) and then through the sensor test chamber together with the fecal VOCs,
(iii) 5 min of continuous N_2_ flow passed at 5 L/min flow rate through the sensor test chamber for purging purposes after the sample measurement.

During the entire measurement process, the sensors were successively operated at 8 V for 10 s in a switching mode. Thus, during the operation period of one of the sensors, the other ones remained inactive by not supplying an operation voltage to them. Therefore, all sensors were once active every 100 s, implying 30 s of operation of each sensor during the 5 min duration of each measurement cycle.

**Figure 3:**
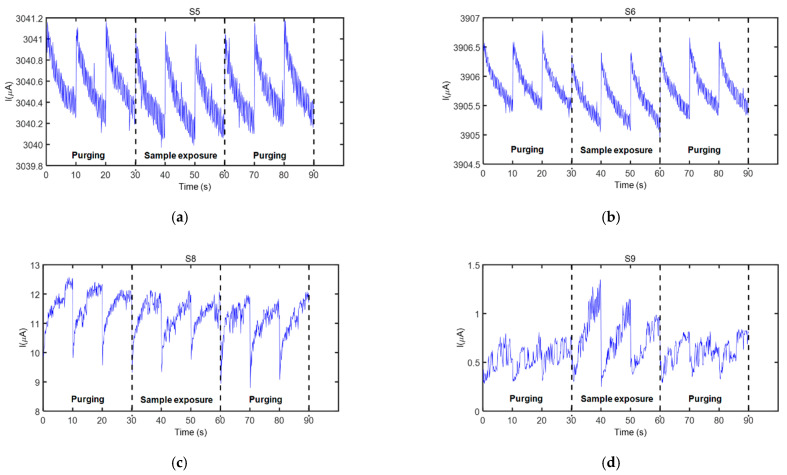
Sensor response (current) as a function of sampling time showing all measurement cycles of the sensors that responded to the fecal VOC samples, when the electronic nose was exposed to one of the samples from wild boar #2 (see Table 1): (**a**) Sensor S5, (**b**) Sensor S6, (**c**) Sensor S8, and (**d**) Sensor S9. Sensor details are found in Table 3.

A high precision power source (B2902A, Keysight Technologies, Hungary—Figure 2a(C)) controlled the sensor supply, as well as the thermal desorption unit. The dc current through each sensor during each operation period was acquired at nine samples/s acquisition rate employing a high resolution data acquisition system (34992A LXI/Data Acquisition Keysight Technolgies, Hungary—Figure 2a(B)) and stored in a computer (Figure 2a(A)) for further analysis. The measurement process consisted of three complete operation periods per sensor for each 5-min measurement cycle (see Figure 3).

### 2.3. Data Analysis

**Figure 4:**
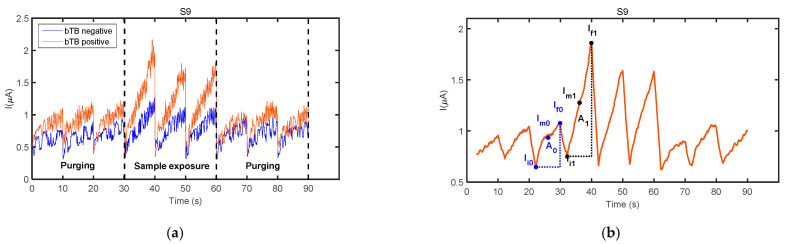
(**a**) Exposure of sensor S9 to the fecal VOC samples of a bTB negative wild boar (blue curve—boar #2 in Table 1) and a bTB positive wild boar (red curve—boar #12 in Table 1). (**b**) Parameters used to calculate sensor response features, indicated on the filtered signal of sensor S9 to the fecal VOC sample of wild boar #12. Sensor details are found in Table 3.

A 20-point moving average filter was applied to the acquired sensor signals in order to reduce the noise generated by the electronic devices. Three features were extracted on the filtered signals from sensor responses to each sample analyzed (see Figure 4b).

- F1: A_1_/A_0_, where A_1_ is the area under the curve calculated from the first 70 data points of the filtered signal for the first operation period of the sensor exposure to the fecal VOC sample, and A_0_ is the area under the curve calculated from the first 70 data points of the filtered signal obtained in the last operation period of the same sensor during the purging process immediately prior to sample exposure,
- F2: (I_m1_ − I_m0_)/I_m0_, where I_m1_ is the averaged current calculated with the first 70 current values of the filtered signal for the first operation period of the sensor exposure to the fecal VOC sample, and I_m0_ is the average current calculated with the first 70 current values of the filtered signal obtained in the last operation period of the same sensor during the purging process immediately prior to sample exposure,
- F3: (∆I_1_ − ∆I_0_)/∆I_0_, where ∆I_1_ = I_f1_ − I_i1_ and ∆I_0_ = I_f0_ − I_i0_, and where I_f1_ and I_i1_ are the 70th and 1st current values, respectively, of the filtered signal for the first operation period of the sensor exposure to the fecal VOC sample, and I_f0_ and I_i0_ are the 70th and 1st current values, respectively, of the filtered signal obtained in the last operation period of the same sensor during the purging process immediately prior to sample exposure.

One of the two samples collected from each wild boar was employed for building classification models for bTB positive and bTB negative animals. The Discriminant Function Analysis (DFA) pattern recognition algorithm was used for this aim [26]. The analysis was separately performed for each age group of animals: adult, sub-adult, and juvenile. The selection of the most discriminative sensors and features for building the classification models for each age group was heuristically achieved by calculating the discrimination accuracy of the models through leave-one-out cross-validation, which is an error estimation that was extensively employed in the literature when analyzing small sample biological datasets [27]. Only the sensors that responded to the fecal VOC samples (see the Results section) were considered for building the classification models. All possible combinations of at least two features extracted from these sensors were assessed, and those that achieved the best classification result for each age group were finally selected. The discrimination potential of the models built was verified by assessing the classification accuracy of the second sample of those animals for which two samples were available (see Table 1).

## 3. Results

### 3.1. Sensor Responses

Four sensors from the electronic nose employed in this study responded to the fecal VOC samples analyzed. These sensors were S5, S6, S8, and S9 (see Table 3 for details), and their characteristic responses to a representative VOC sample are presented in Figure 3. Only the three operation periods in the active mode of the sensors, acquired during each measurement cycle, are indicated in this figure (initial purging process (first three operation periods), sample exposure (next three operation periods), and purging process after a sample measurement (last three operation periods)). Current modulation during the 90 data points acquired during every operation period of a sensor can be clearly observed in this figure.

Sensors’ exposure to the fecal VOC samples of bTB positive and bTB negative boar revealed slightly different response patterns, as visible in Figure 4a. In general, a good repeatability between the three operation periods of the same measurement cycle was observed for all sensors and samples analyzed, as denoted by sensors’ responses presented in Figure 3 and Figure 4. However, in some cases, a drift was noticed during the three operation periods of sample exposure, as it was the case presented in Figure 3d. For this reason, only the first operation period of the sensors acquired during sample exposure and the last operation period acquired during the purging process immediately prior to sample exposure were considered for feature extraction. The parameters used to calculate sensor response features are presented in Figure 4b.

### 3.2. Classification Results

**Figure 5:**
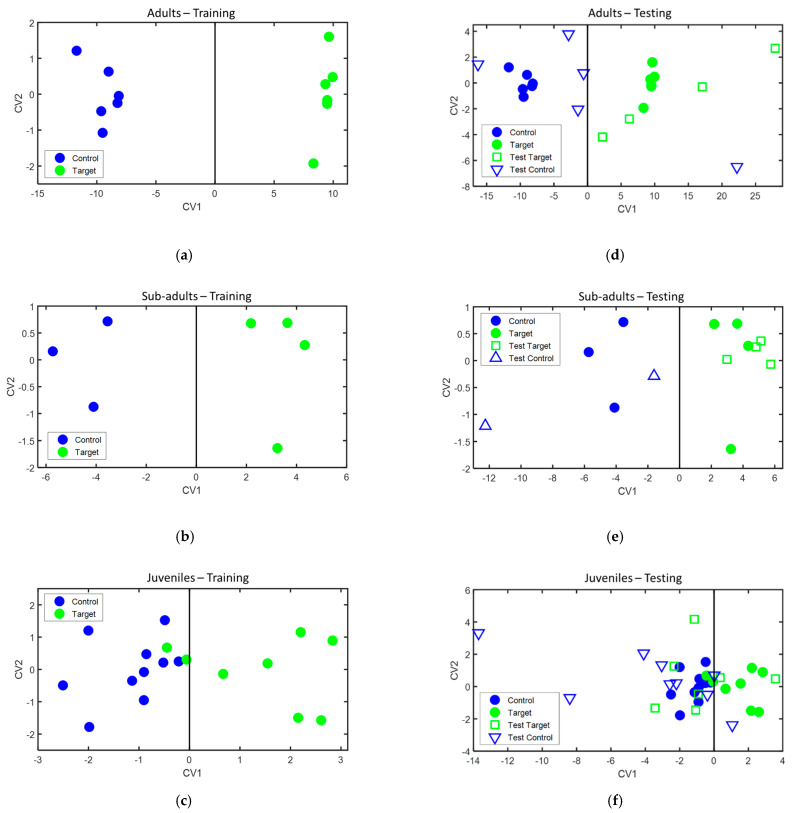
Classification models built for: (**a**) adult animals, (**b**) sub-adult animals, and (**c**) juvenile animals. Projection of the test samples on the classification models for: (**d**) adult animals, (**e**) sub-adult animals, and (**f**) juvenile animals. CV1 and CV2: canonical variables of the DFA model, calculated as a linear combination of sensor features, as described in Ionescu et al. [26]. Legend: control—bTB negative animals, target—bTB positive animals, filled symbols—samples used in the training phase, and empty symbols—samples used in the testing phase.

During the training phase, classification models were separately built for each age group of animals with one sample acquired from each animal. The classification models obtained are presented in Figure 5 (left panels), while the sensors and features used to build each one of the models are presented in Table 4. Leave-one-out cross-validation of these models provided 100% accuracy, 100% sensitivity, and 100% specificity in the case of the adult and sub-adult animals, and 88.9% accuracy, 75% sensitivity, and 100% specificity in the case of the juvenile animals.

During the testing phase, when the second sample of those animals for which two samples were available, was projected over the classification models built during the training phase. Their classification achieved 88.9% accuracy, 100% sensitivity, and 80% specificity for the adult animals, 100% accuracy, 100% sensitivity, and 100% specificity for the sub-adult animals, and 62.5% accuracy, 28.6% sensitivity, and 88.9% specificity for the juvenile animals. Their projection on the classification models is presented in Figure 5 (right panels), and the classification results during both the training and testing phase are summarized in Table 5.

## 4. Discussion

For performing this study, we built an electronic nose system comprising an array of cross-reactive chemical gas sensors based on organically-functionalized gold nanoparticles. This kind of sensors demonstrated very good suitability for detecting low VOC concentrations in biological samples, and room temperature operation that is a key feature of these nanomaterials [28,29,30].

The organic ligands were carefully selected with the intent to present affinity to a broad range of VOCs that are generally found in fecal samples, such as aromatic compounds, alcohols, ketones, esters, aldehydes, alkanes, alkenes, and furans [14,16,17]. The sensing materials were deposited by AGD, which allows for the fabrication of ultra-pure nanomaterials [31]. Sensor resistances ranged between several kΩ up to several MΩ, which are ideal values for gas sensing applications.

Animals captured at different sites located on a large area of 4.4-km radius inside Doñana National Park were included in this study in order to deal with possible sources of contamination present in a national park that could influence the composition of feces (e.g., natural sources of watering and contact with other wild animals).

The sensors that presented good responses to the fecal VOC samples analyzed in this study were sensors S5, S6, S8, and S9 (see Table 3 for details). S5 and S8, with organic ligands with an aromatic functional group in their tail, are likely to have high affinity for VOCs with aromatic functional groups in their structure, while the organic ligand of S6, with a long chain polar group in its tail, has good affinity for polar molecules, such as ketones. This feature is in line with a previous study in which aromatic compounds and ketones were reported as tentative fecal VOC biomarkers for bTB in swine [17]. S9, which employed 1-butanethiol as an organic ligand, has high affinity for non-polar VOC molecules, such as alkanes and alkane derivatives, and certainly widened the range of detected VOCs.

Yet, other sensors had similar organic functionalities as the sensors that responded to the fecal VOC samples analyzed (e.g., S7 and S8, or S1 and S5, respectively). However, they could present very different morphologies because of slightly different experimental conditions during their fabrication [25]. This is indicated by their different resistance values presented in Table 3. Therefore, their detection potential for fecal VOCs may not have been totally tuned.

The dynamic sensors’ operation mode employed in this study, implemented by applying short voltage pulses to the sensors (10 s duration) followed by longer inactivity periods (90 s duration), allowed for analyzing the surface adsorption-desorption reaction kinetics that occur between the sensing material and the sensed VOCs. This operation mode provides more information than the static operation mode when the sensor is continuously operated at a constant voltage [32].

During the short voltage pulses when the sensor was in the active state, the current through the sensor varied between two values, which either decreased or increased, depending on the organic functionality linking the gold nanoparticles (see sensor responses presented in Figure 3 and Figure 4). It is important to note that the sensors did not achieve a steady state condition (i.e., constant stabilized current) during their operation, which ensured that only their dynamic operation was assessed in this study. Considering the sensors that presented a good response to the fecal VOC samples analyzed, for 2-mercaptobenzoxazole and 11-mercaptoundecanoic acid, the current decreased during the voltage supply, which produces a slight heating effect, suggesting a p-type semiconducting behavior, while for 4-methoxy-α-toluenethiol and 1-butanethiol, the current increased during the voltage supply, suggesting an n-type semiconducting behavior.

For data analysis, only the first operation period of each sensor during VOC sample exposure was considered for extracting sensor features, corresponding to the first 100 s of VOC exposure, as each sensor was successively active for 10 s during this operation period. Actually, it is expected that a higher concentration of the VOCs desorbed in the thermal desorption unit was transferred by N_2_ flow to the sensor test chamber in the first moments of sample exposure. Figure 3d revealed that even S9, the penultimate activated sensor, presented a noticeable response to the VOC sample during its first operation period, suggesting that VOC concentration was still present in a suitable level in the measurement chamber.

The classification models built based on sensor responses showed very good accuracies for bTB detection for the adult and sub-adult animals, while, for the juvenile animals, the results were not adequate (see Table 5). Following previous analytical studies that suggested an age-dependent difference of the metabolomics pathway of bTB in swine [17], different sensor features were chosen in this study to build more robust bTB classification models for the different age groups (see Table 4).

The adult and sub-adult animals were sedated with a combination of tiletamine-zolazepam and medetomidine, which avoided their stress during sample collection while not affecting VOC composition, as reported in our previous study [17]. The juvenile animals were manually restrained while collecting the fecal sample, which may have induced a stress response, possibly altering the composition of the pattern of VOCs emitted by biological samples [17]. However, other metabolic factors in juveniles could have made their VOCs take on a different pattern as well. In order to clarify this point, further research should be done by either sedating the juvenile animals in a similar manner to the adult and sub-adult animals while collecting the fecal samples, or by passively collecting the samples without handling them.

The influence of the number of animals from each age group on the results obtained cannot be disregarded. The classification of the sub-adult animals could be easier because of the smaller size of this age group. The larger sample size of the juvenile group could have complicated their classification with the DFA algorithm employed in this study. Other pattern recognition algorithms that could better adjust to the dataset of the juvenile animals could be assessed in order to try to obtain better results.

Overall, the present study complements the current research trend focused on the investigation of alternative methods based on the measurement of VOCs emitted by biological samples with electronic nose systems for simpler and less expensive diagnosis of diseases. While numerous studies of this kind were realized on human beings, the studies realized on animals are rather scarce [12]. Actually, the present study is the first report that assesses the diagnosis of an animal disease from the measurement of the VOCs emitted by feces with an electronic nose system. For *M. bovis* diagnosis in bovines, promising results were obtained in the analysis of breath samples with the NA-NOSE electronic nose system developed by Technion Israel Institute of Technology comprising an array of six chemical gas sensors based on organically-functionalized gold nanoparticles (86.4% accuracy on a population of 22 animals) [20], as well as in the analysis of the VOCs emitted by serum samples with the commercial αFOX3000 MOS electronic nose system developed by Alpha M.O.S. Co. (Tolouse, France) based on an array of nine metal oxide semiconducting gas sensors (95.2% accuracy on a population of 21 animals) [19].

## 5. Conclusions

This research reports a proof-of-concept study regarding the diagnosis of bTB, caused by *Mycobacterium bovis*, in wild boar, from the analysis of the VOCs emitted by animal feces with an electronic nose system. The analysis of fecal samples was selected because they can be non-invasively collected and have great potential to provide a convenient means for the detection of *M. bovis* in wildlife populations.

The study was realized on free-ranging wild boar captured in Doñana National Park, Spain, where bTB disease is endemic in ungulates. In order to counteract the influence of possible confounding factors on feces’ composition (e.g., natural sources of watering, contact with other wild animals), animals captured on a large area of a 4.4-km radius inside the park were included in the study.

The samples were analyzed with a home-made electronic nose system that comprised an array of 10 chemical gas sensors based on organically-functionalized gold nanoparticles fabricated employing the AGD technique, which yields ultrapure and narrow size-ranged nanoparticle synthesis. During sample measurement, the sensors were operated in the dynamic mode by successively applying short voltage pulses to each one of them in a switching mode, which allowed for investigating the reaction kinetics that occur between the sensing film and the VOC sample.

The study was separately performed on three age groups of animals: adult, sub-adult, and juvenile. The classification results built with the DFA pattern recognition algorithm provided 100% accuracy during both model training and validation for the sub-adult animals, 100% accuracy during model training and 88.9% during model validation for the adult animals, and 88.9% accuracy during model training and 62.5% during model validation for the juvenile animals. The obtained results could have been influenced by the sample size of each age group (smaller in sub-adult and larger in juvenile animals), as well as animal handling during sample collection (the sub-adult and adult animals were anesthetized while the juveniles were manually restrained).

Overall, while taking into account the necessity to handle all animals similarly in future studies, the results obtained in this study could pave the way for the further development and implementation of a non-invasive and less expensive approach to detect bTB disease in wild populations. Nevertheless, the reported results need to be validated on larger cohorts of animals, while other pattern recognition techniques could be assessed to explore algorithms that may more effectively adjust to the dataset of juvenile animals.

## 6. Patents

A patent application was filed with the results reported in this manuscript. Title: In vitro methods for the diagnosis of bovine tuberculosis in swine; Applicant: The United States as Represented by the Secretary of Agriculture, Washington, DC; Inventors: Radu Ionescu and Pauline Nol; Application number: US62/981,591; Filing date: 02/26/2020.

## Figures and Tables

**Table 1:** Information about animals and samples analyzed in this study.

Animal No.	*M. bovis* Infection	Sex	Age ^1^	Location	Number of Samples ^2^
1	Positive	Male	Adult	Martinazo	2
2	Negative	Male	Juvenile	Santa Olalla	2
3	Positive	Male	Juvenile	Palacio	2
4	Positive	Male	Juvenile	Palacio	2
5	Positive	Female	Adult	Palacio	2
6	Positive	Female	Sub-adult	Palacio	2
7	Negative	Male	Sub-adult	Santa Olalla	2
8	Negative	Female	Sub-adult	Santa Olalla	2
9	Positive	Male	Adult	Santa Olalla	2
10	Negative	Male	Juvenile	Santa Olalla	2
11	Negative	Female	Adult	Santa Olalla	2
12	Positive	Male	Adult	Santa Olalla	2
13	Negative	Male	Adult	Santa Olalla	2
14	Negative	Female	Adult	Martinazo	2
15	Positive	Female	Juvenile	Martinazo	2
16	Negative	Male	Juvenile	Martinazo	2
17	Positive	Male	Sub-adult	Martinazo	2
18	Positive	Male	Sub-adult	Martinazo	2
19	Negative	Female	Juvenile	Palacio	2
20	Negative	Female	Juvenile	Palacio	2
21	Negative	Female	Juvenile	Martinazo	2
22	Negative	Female	Juvenile	Santa Olalla	2
23	Negative	Female	Adult	Santa Olalla	2
24	Negative	Female	Juvenile	Santa Olalla	2
25	Positive	Female	Juvenile	Martinazo	2
26	Positive	Male	Juvenile	Martinazo	2
27	Positive	Female	Juvenile	Martinazo	2
28	Positive	Female	Juvenile	Martinazo	1
29	Positive	Male	Adult	Santa Olalla	1
30	Positive	Female	Adult	Santa Olalla	1
31	Positive	Male	Juvenile	Martinazo	2
32	Negative	Female	Adult	Martinazo	1
33	Negative	Male	Adult	Santa Olalla	2
34	Positive	Male	Sub-adult	Santa Olalla	2
35	Negative	Female	Juvenile	Santa Olalla	2
36	Negative	Male	Juvenile	Fuente del Duque	1
37	Negative	Female	Sub-adult	Martinazo	1

^1^ Adult: >24 months, Subadult: 12–24 months, Juvenile: <12 months. ^2^ The second VOC sample was collected from the same fecal sample introduced in the same jar, immediately after the collection of the first sample. In some cases, one of the samples was damaged during transport and/or handling. Therefore, for some animals, only one sample was available for the analysis.

**Table 2:** Animal grouping on age, disease, sex, and location.

Age	Location	bTB Negative		bTB Positive	
		Male	Female	Male	Female
Adult	Santa Olalla	2	2	3	1
	Martinazo	-	2	1	-
	Palacio	-	-	-	1
	Total	2	4	4	2
Sub-adult	Santa Olalla	1	1	1	-
	Martinazo	-	1	2	-
	Palacio	-	-	-	1
	Total	1	2	3	1
Juvenile	Santa Olalla	2	3	-	-
	Martinazo	1	1	2	4
	Palacio	-	2	2	-
	Fuente del Duque	1	-	-	-
	Total	4	6	4	4

**Table 3:** Information about the sensors employed in this study.

Sensor No.	Organic Functionality	Electrical Resistance
S1	2-Mercaptobenzoxazole	347 kΩ
S2	Methyl-3-mercaptopropionate	253 kΩ
S3	1-Decanethiol	506 kΩ
S4	1-Decanethiol	641 kΩ
S5	2-Mercaptobenzoxazole	1.5 kΩ
S6	11-Mercaptoundecanoic acid	1.6 kΩ
S7	4-Methoxy-α-toluenethiol	11 kΩ
S8	4-Methoxy-α-toluenethiol	6.8 MΩ
S9	1-Butanethiol	759 kΩ
S10	Octadecylamine	6.2 MΩ

**Table 4:** Sensors and features used to build the classification models for each age group of animals. In this table, “a” represents adult, “s” represents sub-adult, and “j” represents juvenile. Sensor details are presented in Table 3, and sensor features in Section 2.3.

		Sensors			
		S5	S6	S8	S9
Features	F1	-	a, s	a, j	-
	F2	a, j	s	s, j	j
	F3	a, j	j	a, s, j	j

**Table 5:** Success rates achieved by the classification models during the training and testing phases, respectively. TN: true positives. TN: true negatives. FP: false positives. FN: false negatives. Accuracy = (TP + TN)/(TP + TN + FP + FN). Sensitivity = TP/(TP + FN). Specificity = TN/(TN + FP).

Phase	Age Group	Accuracy (%)	Sensitivity (%)	Specificity (%)	TP	TN	FN	FP
Training	Adult	100	100	100	6	6	0	0
	Sub-adult	100	100	100	4	3	0	0
	Juvenile	88.9	75	100	6	10	2	0
Testing	Adult	88.9	100	80	4	4	0	1
	Sub-adult	100	100	100	4	2	0	0
	Juvenile	62.5	28.6	88.9	2	8	5	1

## Data Availability

The data presented in this study are available on request from the corresponding author. The data are not publicly available due to patenting issue.
